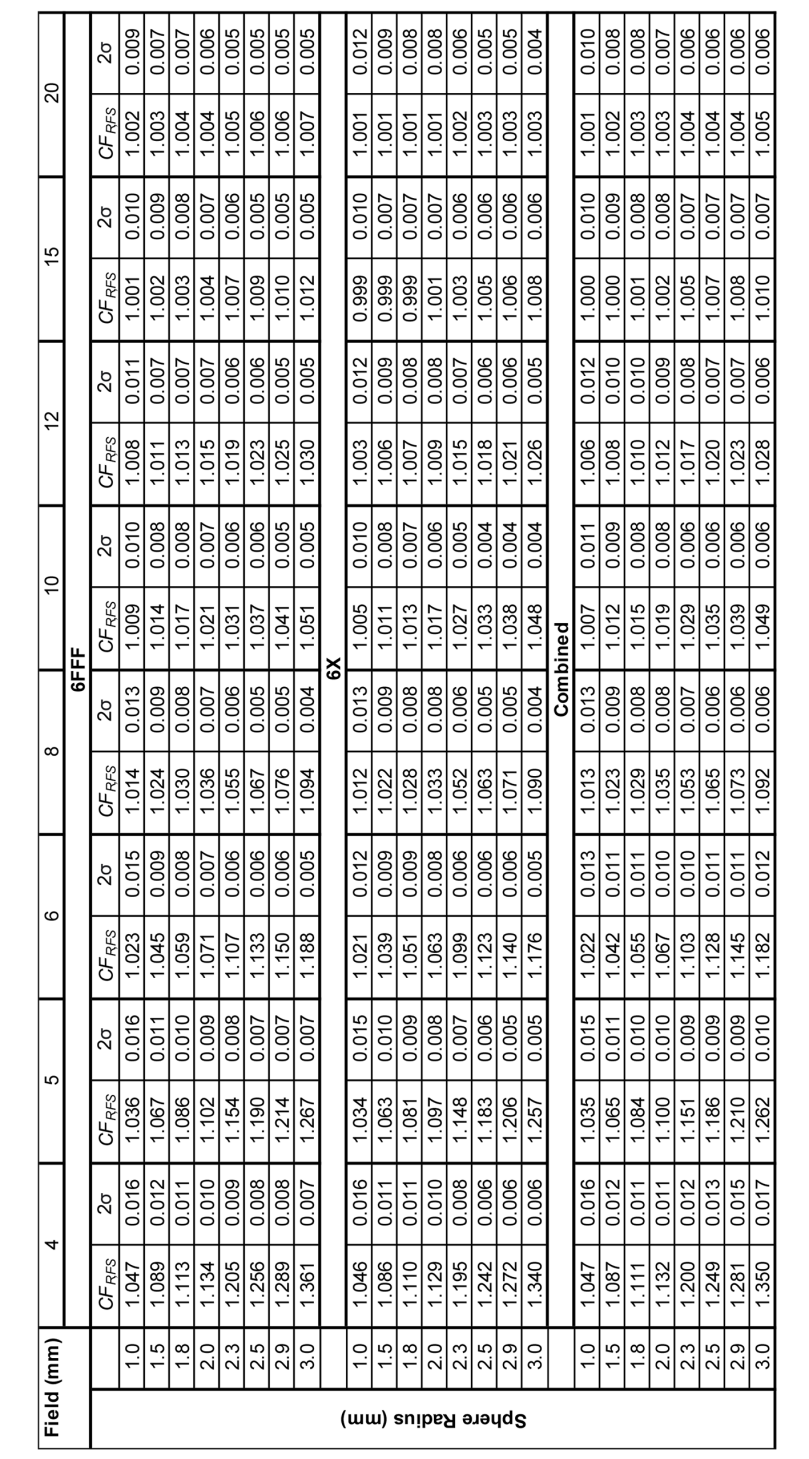# Correction factors for commissioning and patient specific quality assurance of stereotactic fields in a Monte Carlo based treatment planning system: TPS correction factors

**DOI:** 10.1007/s13246-023-01246-3

**Published:** 2023-04-06

**Authors:** Simon K. Goodall, Pejman Rowshanfarzad, Martin A. Ebert

**Affiliations:** 1 School of Physics, Mathematics, and Computing, The University of Western Australia, Crawley, WA 6009 Australia; 2 GenesisCare, 24 Salvado Road, Wembley, WA 6014 Australia; 3 Department of Radiation Oncology, Sir Charles Gardiner Hospital, Nedlands, WA 6009 Australia; 4 5D Clinics, Claremont, WA 6010 Australia

**Keywords:** Patient Specific Quality Assurance, PSQA, SRS QA, SBRT QA, Correction factors, Ionisation chamber measurments

## Abstract

Validation of small field dosimetry is crucial for stereotactic radiosurgery (SRS) and stereotactic body radiotherapy (SBRT). Accurate and considered measurement of linear accelerator dose must be compared to precise and accurate calculation by the treatment planning system (TPS). Monte Carlo calculated distributions contain statistical noise, reducing the reliance that should be given to single voxel doses. The average dose to a small volume of interest (VOI) can minimise the influence of noise, but for small fields introduces significant volume averaging. Similar challenges present during measurement of composite dose from clinical plans when a small volume ionisation chamber is used. This study derived correction factors for VOI averaged TPS doses calculated for small fields, allowing correction to an isocentre dose following account for statistical noise. These factors were used to determine an optimal VOI to represent small volume ionisation chambers during patient specific quality assurance (PSQA). A retrospective comparison of 82 SRS and 28 SBRT PSQA measurements to TPS calculated doses from varying VOI was completed to evaluate the determined volumes. Small field commissioning correction factors of under 5% were obtained for field sizes of 8 mm and larger. Optimal spherical VOI with radius between 1.5 and 1.8 mm and 2.5 to 2.9 mm were determined for IBA CC01 and CC04 ionisation chambers respectively. Review of PSQA confirmed an optimal agreement between CC01 measured doses and a volume of 1.5 to 1.8 mm while CC04 measured doses showed no variation with VOI.

## Introduction

Validating the ability of a Treatment Planning System (TPS) to accurately calculate small field dose is a key step during the commissioning of Stereotactic Radiosurgery (SRS) and Stereotactic Body Radiotherapy (SBRT). [1–3] This is a two-part process; first accurate measurement of small field dose must be completed, second, the dose must be calculated both accurately and precisely within the TPS to allow comparison. Measurement of small field dosimetry carries significant challenges which are well documented, and a number of guidelines are available. [3–5] Precise calculation of dose in small fields within a TPS is generally achievable if care is taken and calculations are completed with sufficient resolution.

The use of Monte Carlo (MC) algorithms, commonly considered the gold standard for radiotherapy treatment planning, is increasing in clinical practice. [6,7] Dose distributions calculated using these algorithms inherently contain statistical noise which can present challenges in the determination of dose at a highly specific location. Guidelines warn against the consideration of dose calculated for a specific voxel and instead recommend calculation of an average dose over a number of surrounding voxels, or a Volume of Interest (VOI), to minimise the impact of statistical noise. [6,8] For a given location, the Monaco v6.00 (Elekta AB, Stockholm, Sweden) MC TPS displays both the voxel dose and the mean dose to a sphere of a user defined radius to allow extraction of this data. This however introduces challenges during the commissioning process, where calculations are required to have high spatial and dosimetric precision.

The ability to use a VOI mean dose is particularly challenging for small field calculations due to the limited number of voxels within the treatment field and the potentially significant dose gradients across the in-field profile. To avoid the need for a VOI an increasingly high number of MC histories are required which is time consuming and may not be possible in some commercial systems, such as Monaco, which have a limit on the number of histories that can be used. [8] An alternate approach would be to use correction factors applied to VOI average doses, such as those used during measurements however the required factors are not well established for TPS calculations. [3].

Following the evaluation of measured and calculated small static field doses, ionisation chamber measurements are commonly used, often in combination with film or diode arrays, during the validation of composite treatment plan deliveries. [9–12] This can be during end-to-end testing, often the final stage of commissioning, or during the Patient Specific Quality Assurance (PSQA) of clinical plans. SRS and SBRT plans inherently target small volumes using steep dose gradients and therefore require measurement using small sensitive volume ionisation chambers.

During these PSQA workflows similar small field dosimetry challenges present. There is a need to calculate dose precisely at a clinically relevant location and a need to measure dose as a composite of a very high number of small fields, or beam segments, all of different shape and equivalent square size. Due to the continuously varying field sizes used in Volumetric Modulated Arc Therapy (VMAT) or Intensity Modulated Radiotherapy (IMRT) it is not possible to directly apply those published correction factors used for the measurement of small fields. [3,13] Instead, a VOI representing the ionisation chamber within the TPS is often used to derive the expected measured dose. [14,15] As a result, volume averaging is applied to both the calculation and the measurement, which allows for direct comparison when assuming the effects are equal in both circumstances. For MC calculations it is also important that the VOI is sufficiently large to account for statistical noise, which can be problematic when using small field ionisation chambers.

This study aimed to derive a series of correction factors which could be applied to VOI averaged small field doses, as calculated within a TPS, to allow the determination of the central single voxel dose. Subsequently it aimed to determine the magnitude of volume averaging that should be applied to obtain reliable results from a single calculation, whilst minimising the magnitude of the applied correction factor. Finally, it aimed to determine the volume over which averaging in the TPS should be applied during PSQA to obtain best agreement when composite plan measurements are made using an ionisation chamber.

## Methods

### Correction factor derivation

A series of 40 different water phantoms with dimensions greater than 20 cm in all directions were artificially created using MIM v7.1.4 (MIM software Inc) and exported to Monaco. Separate phantoms are required in the Monaco TPS to produce calculations using different initial seed points. [8] Two separate plans were created for each phantom, one using 6MV and the other 6FFF. Each plan consisted of square fields with side length varying between 4 and 20 mm, used a 90 cm SSD and an isocentre at depth of 10 cm. The per beam dose distributions were calculated using an isotropic dose voxel of 1 mm and a statistical uncertainty of 1.0% per control point. The plan and RT dose file were then exported for analysis using MATLAB R2021b (Mathworks, Natick, MA).

The isocentre voxel dose was extracted along with the mean dose to a range of spheres, centred about the isocentre and with varying radius, for each beam of each plan. Monaco uses a Boolean logic to determine if a voxel is included in the calculation of the sphere mean dose and radii were therefore selected as those at which additional voxels became included in the calculation of the mean, as documented in Table 1. The use of 40 phantoms resulted in the confidence interval for the isocentre dose being ≤ 0.5% at the 95% level and ≤ 0.7% at the 99% level for all field sizes.

**Table 1:** The radii, volume and number of voxels contained in the spherical volumes of interest considered

Sphere Radius (mm)	True Volume (mm [3])	Included Voxels (mm [3])
1.0	4.2	7
1.5	14.1	19
1.8	24.4	27
2.0	33.5	33
2.3	51.0	57

The true TPS calculated isocentre doses for a given energy and field size (FS), *D*_*I,FS*_, were determined as the mean of the 40 extracted isocentre voxel dose values taken from each phantom calculation for a given energy. Similarly, for a given sphere radius (R), the true TPS calculated mean sphere dose, *D*_*R, FS*_, was determined as the mean of the 40 extracted sphere doses, for equal sized spheres across all phantoms. The correction factor, *CF*_*R,FS*_, required to determine the TPS calculated isocentre dose from the TPS calculated sphere dose was then calculated for each field size and sphere radius combination as

$$ {CF}_{R,FS}=\frac{{D}_{I,FS}}{{D}_{R,FS}}$$

The values of *CF*_*R,FS*_ were finally averaged across the two beam energies due to the similarity of the derived factors (see appendix 1).

### Correction factor uncertainty

The relevant derived factor was retrospectively applied to the extracted sphere doses from each individual beam, *D*_*R,FS,B*_. The error in the corrected sphere dose for each beam of each plan, *Error*_*B*_, relative to the true TPS calculated isocentre dose was calculated as

$$ {Error}_{B} \left(\%\right)= \frac{{CF}_{R,FS}{D}_{R,FS,B}}{{D}_{I,FS}}-1$$

For every field size and sphere radius combination, the standard deviation of the 80 error values was determined to establish a measure of uncertainty in the derived factors to individual beam calculations.

### Sphere radius association with ionisation chambers

In addition to the sphere doses determined by Monaco, structure-based models of CC01 and CC04 ionisation chambers (IBA dosimetry, Schwarzenbruck, Germany) were considered. The CC01 and CC04 chambers have inner radii of 1.0 and 2.0 mm respectively and both have an active length of 3.6 mm, generating their 0.01 cc and 0.04 cc volumes. [16] Inclusion of dose voxels by given structures within Monaco is determined via consideration of the CT resolution and CT voxel size. [17] Manual contouring of a volume equivalent to the sensitive volume of an ionisation chamber at the plan isocentre will therefore result in varied inclusion of the surrounding dose voxels on an inter-CT basis. To ensure consistency during this investigation, mean doses to a shape equivalent to the sensitive volume of a CC01 and a CC04, centred on the isocentre (hence the central dose voxel) [17] were extracted using a MATLAB script. These volumes differed from the inbuilt VOI used by Monaco by allowing non spherical shaping and partial voxel inclusion via a weighted mean dose calculation. Correction factors were calculated for the ionisation chamber models using the same convention described in Sect. Correction factor uncertainty.

To determine the sphere radius, or chamber model, which best represented an ionisation chamber under PSQA measurement conditions, the TPS correction factors calculated as per Sect. Correction factor uncertainty were compared to the correction factors presented by the IAEA for use during output factor measurement. [3] During measurement the IAEA factors are applied to determine a corrected point dose, in the form of an output factor. Similarly, the correction factors calculated as per Sect. Correction factor uncertainty determine the corrected point dose from a small VOI within the TPS. For an ideal representation of an ionisation chamber within the TPS, the IAEA and TPS correction factors could therefore be expected to be equal for like field sizes. As such, the sphere radius requiring correction factors most similar to the IAEA factors, over a range of small field sizes, was hypothesised to best represent the ionisation chamber performance. The use of this sphere size for determination of the TPS calculated dose during PSQA was therefore expected provide best agreement with measurement when no correction factors are applied to the measured values.

### Comparisons to PSQA

The agreement between PSQA ion chamber measurements and Monaco calculated doses were assessed for the range of considered sphere sizes and the chamber models. PSQA measurements were made over five different Elekta Versa HD linacs (Elekta AB, Stockholm, Sweden) situated over three different states of Australia. All linacs have previously been shown to be well matched to a single beam model within the Monaco TPS and to each other. [18] A total of 82 cranial SRS treatment plans with either single or multiple targets per isocentre, using between 3 and 6 non coplanar VMAT arcs and a 6FFF energy, were measured using a CC01 ionisation chamber in a solid water phantom. A total of 28 SBRT prostate, bone or lung plans using coplanar dual VMAT arcs and a 6FFF energy were measured using a CC04 ionisation chamber in either a solid water phantom or the CIRS thorax phantom (Sun Nuclear Corporation, Norfolk, VA, USA). All plans were calculated using a 0.1 cm isotropic dose grid and statistical uncertainty of 0.7% per plan.

The Pearson correlation coefficient, sum of squared errors (SSE), mean and median relative error across all measurements were calculated when comparing the measured dose to the set of TPS calculated doses for each sphere size or chamber model. These values were then calculated again considering only the 45 cranial SRS plans (measured using CC01) for which the calculated dose changed by ≥ 1.0% across the considered sphere sizes.

### Identification of sensitive treatment plans

To determine those plans which are most likely to show changes in calculated dose across the considered VOI size, the Planning Target Volume (PTV), Heterogeneity Index (HI) and delivered dose per MU were extracted from the treatment plan. These were compared to variation in the predicted dose across all sphere sizes and chamber models considered.

## Results

### Correction factor values

The derived corrections factors are shown in Fig. 1 for the values averaged across the two beam energies. Larger correction factors are required for decreasing field sizes and increasing sphere radius as would be expected.

**Fig. 1:**
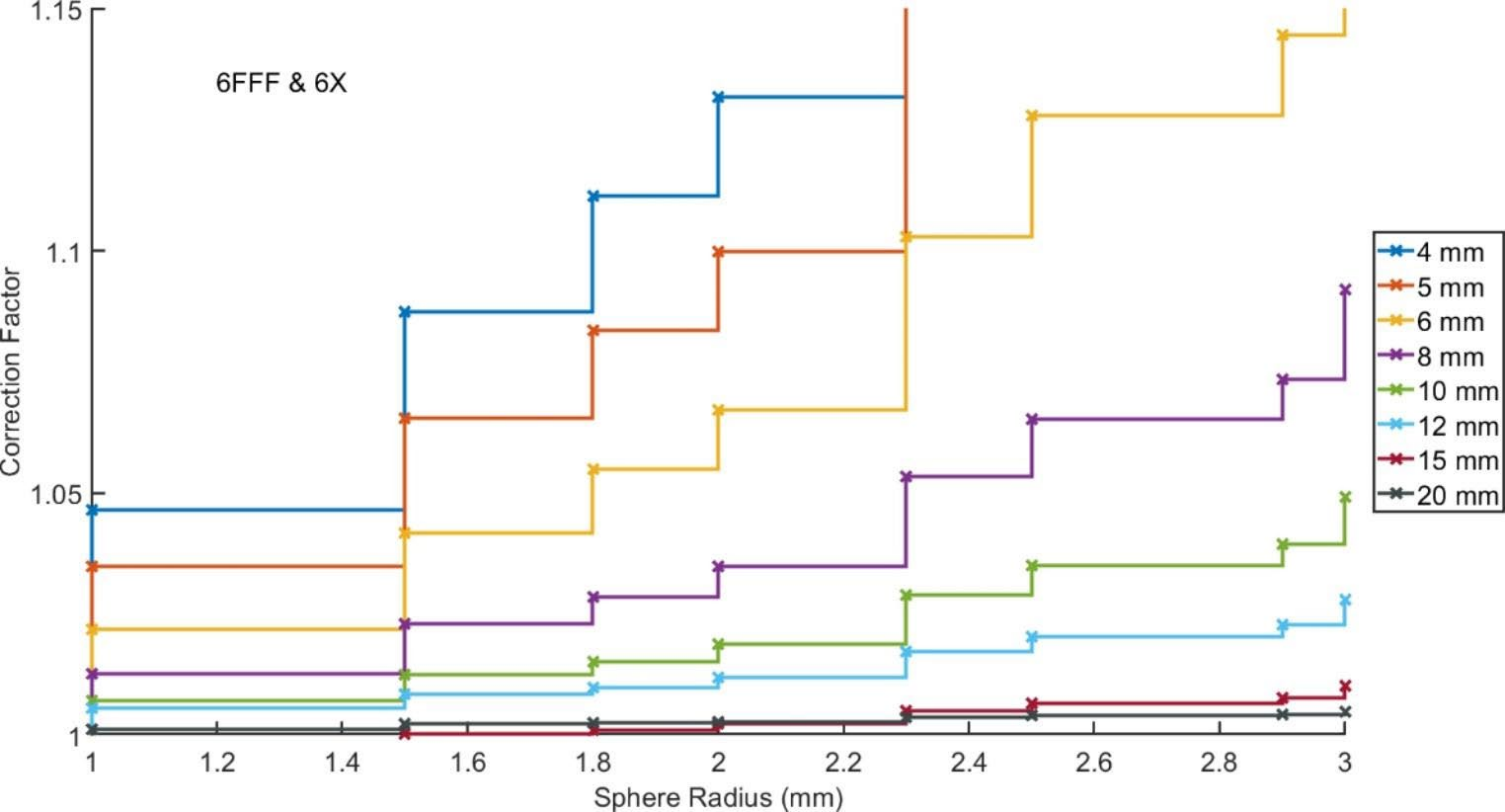
Correction factors for various field sizes and sphere radii. Each line presents the correction factors for a square field of side length given in the legend

The correction factors for the two beam energies were similar, with slightly higher factors required for 6FFF due to the nature of the beam profile. Maximum differences were observed for the smallest field and largest sphere size combination (1.340 v 1.361) and all factors differed by less than 0.01 for field sizes greater than 4 mm or sphere radius less than 3 mm. The full set of factors are tabulated in Appendix 1. A large number of the derived factors for larger spheres or small fields exceeded the maximum suggested correction factor recommended by the IAEA of 5%.^3^ Larger correction factors presented here should therefore not be used in a clinical environment.

### Correction factor uncertainty

Figure 2 shows plots of two standard deviations of the error in the calculated dose at isocentre after application of the factors shown in Fig. 1

**Fig. 2:**
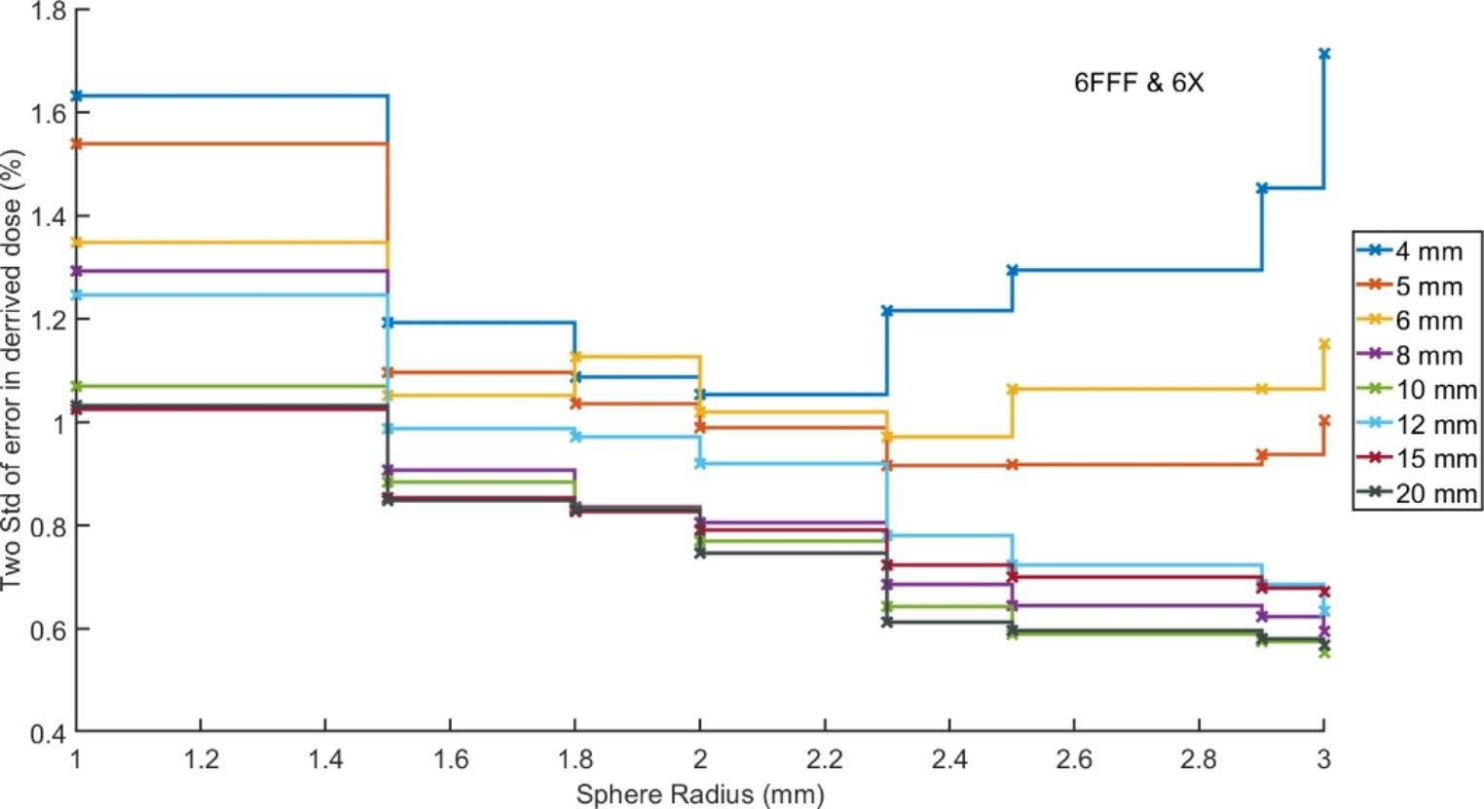
Two standard deviations of the error in isocentre dose as calculated using a mean sphere dose and the correction factors displayed in Fig. 1. Each line presents a different field size.

When using the correction factors shown in Fig. 1, the isocentre dose was calculated to be within 1.0% of the average value obtained across the 80 calculations, for 95% (two standard deviations) of the calculations, when using a minimum sphere size of 1.5 mm for field sizes of 8 mm or greater. For smaller field sizes the correction factors have less predictable results due to the relatively large size of the VOI compared to the field and the averaging across the two beam energies. Minimum values in the standard deviations were observed for the 5 and 6 mm fields for sphere radius of 2.3 mm. It is however important to recognise this radius would encompass the majority of the open field and the correction factors for this radius well exceed 5%. For the 4 mm field the value of two standard deviations never falls below 1%. It is therefore recommended that this approach is not applied, at least alone, for fields sizes under 8 mm, but instead an increased number of histories, or number of phantom based calculations could be completed.

The use of larger sphere sizes reduces the standard deviation of the resultant errors due to averaging over a greater volume of the calculated dose distribution, however it requires the application of a greater correction factor. It is suggested that the smallest sphere, hence smallest correction factor, that provides results with an acceptable accuracy should be used during a commissioning process to minimise the uncertainty contained within the correction factor itself.

### Sphere radius association with ionisation chambers

A comparison of the derived correction factors for a sub-set of sphere sizes plus the chamber models are shown in Fig. 3 alongside the IAEA correction factors for the IBA CC01 and CC04 chambers. [3].

**Fig. 3:**
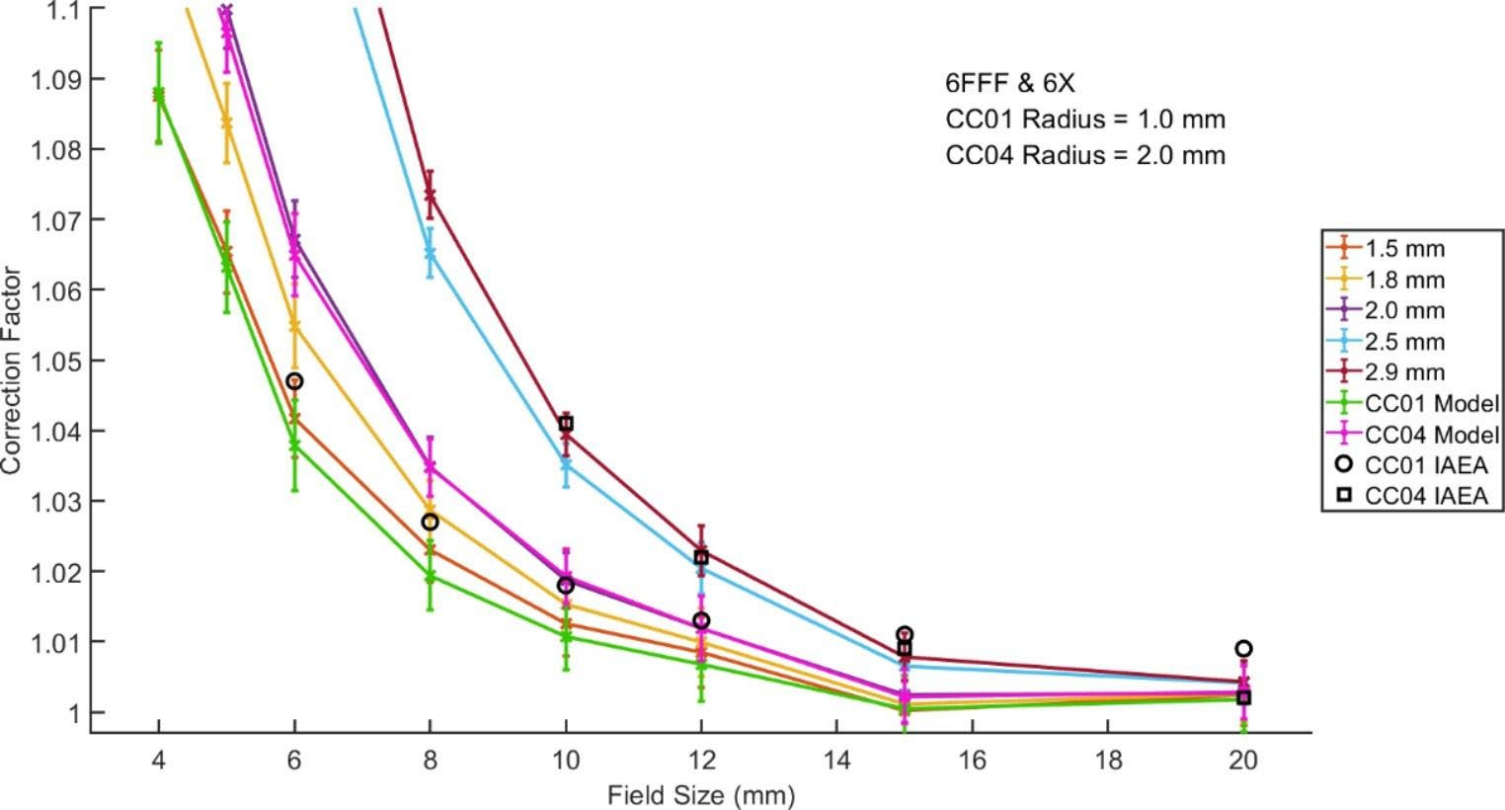
Comparison of the IAEA correction factors for small field output factor measurements and the derived correction factors for various square fields sizes, sphere sizes and chamber models

The correction factors presented by the IAEA are larger than those derived when using chamber models, particularly those for the CC04 model. The IAEA correction factors for the CC01 are most closely matched to spheres of radius between 1.5 and 1.8 mm, those for the CC04 to spheres between 2.5 and 2.9 mm.

Spheres of these radii are approximately one and a half times the sensitive volume of the chambers as described by the manufactures and closer to twice the sensitive volume when considering number of voxels of the dose grid considered by Monaco in the calculation of the mean. These results highlight the fact that the IAEA correction factors include additional corrections to volume averaging and highlight a potential limitation in producing a contour of a chamber to determine the dose expected during a PSQA measurement. [3,13]

### Comparisons to PSQA

Table 2 summarises the calculated comparison values between Monaco and PSQA doses measured using a CC01 and a CC04. Data is presented for all measurements made across a series of 82 SRS measurements and separately for the 45 measurements where the calculated dose was shown to vary by ≥ 1.0% over the range of sphere sizes considered. The data for all 28 SBRT measurements using a CC04 are given, due to the lack of variation in calculated dose across sphere sizes, this data is not further stratified.

**Table 2:** Summary of the calculated comparison values between measured and calculated values for the range of spheres sizes across all clinical plans

Total 82 SRS Measurements (CC01)									
Radius (mm)	1	1.5	1.8	2	2.3	2.5	2.9	3	CC01 model
Correlation	0.9992	0.9992	0.9993	0.9993	0.9993	0.9992	0.9991	0.9989	0.9993
Av Err (%)	0.30	0.13	0.03	-0.06	-0.33	-0.50	-0.63	-0.90	-0.09
Median (%)	0.15	-0.05	-0.14	-0.19	-0.34	-0.63	-0.81	-0.79	-0.24
SSE (%)	1.26	1.23	1.21	1.20	1.21	1.26	1.31	1.46	1.21
Subset of 45 SRS Measurements (CC01)									
Radius (mm)	1	1.5	1.8	2	2.3	2.5	2.9	3	CC01 model
Correlation	0.9992	0.9993	0.9993	0.9993	0.9993	0.9993	0.9993	0.9991	0.9993
Av Err (%)	0.57	0.27	0.09	-0.07	-0.55	-0.88	-1.10	-1.58	-0.12
Median (%)	0.36	0.11	-0.16	-0.54	-1.07	-0.90	-1.24	-1.60	-0.69
SSE (%)	0.78	0.74	0.73	0.73	0.75	0.80	0.86	1.01	0.73
Total 28 SBRT Measurements (CC04)									
Radius (mm)	1	1.5	1.8	2	2.3	2.5	2.9	3	CC04 model
Correlation	0.9988	0.9988	0.9988	0.9988	0.9989	0.9989	0.9989	0.9989	0.9988
Av Err (%)	0.07	0.03	0.04	0.02	-0.05	-0.08	-0.09	-0.12	0.07
Median (%)	-0.28	-0.40	-0.38	-0.45	-0.48	-0.48	-0.50	-0.54	-0.24
SSE (%)	0.35	0.35	0.35	0.35	0.34	0.34	0.34	0.35	0.35

The boxplots in Fig. 4 show the spread of relative dose errors between Monaco and measured PSQA doses for all considered calculation sphere sizes.

**Fig. 4:**
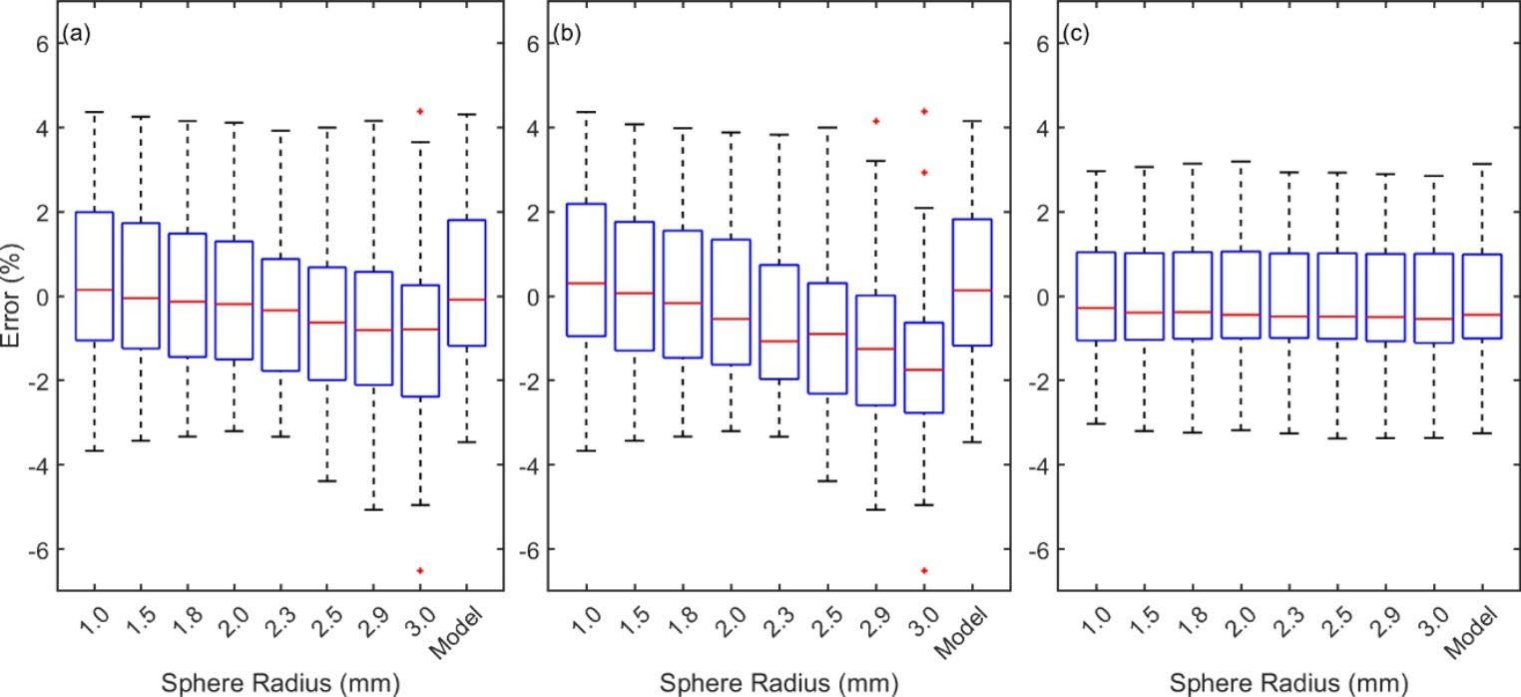
The relative error in measured PSQA compared to Monaco calculated mean doses for spheres of varying radius and a chamber model. All 82 CC01 calculations are considered in boxplot (a) and the 45 calculations which showed a variation of ≥ 1.0% in calculated dose across sphere radii are included in boxplot (b). Boxplot (c) shows the lack of variation for the CC04 calculations

For both the full cohort and subset of plans measured using the CC01, the SSE, mean and median relative errors are closest to zero when using the 1.5 or 1.8 mm sphere radius. This is in agreement with the finding presented in Sect. 3.3. It should be noted however that the median error shows only a small level of variation over the range of spheres, ranging between 0.15% and − 0.81% when considering all measurements, and 0.36% and − 1.60% when considering only those with the highest variations in calculated dose. These ranges are likely less than the uncertainty associated with ionisation chamber PSQA measurements in SRS or SBRT plans. [13,14]

For those plans that were measured using a CC04 a very minimal change in the calculated dose was observed across the measured plans. This is likely due to the larger target volumes used in prostate, bone and lung SBRT, indicating that the choice of sphere size is only important during the measurement of the smallest target volumes which inherently contain the greatest inhomogeneity.

### Identification of sensitive treatment plans

No correlation was observed between the clinical plan PTV heterogeneity or the plan dose per monitor unit and the magnitude of the change in calculated dose across considered sphere radius. Figure 5 shows the variation in calculated dose with the volume of the PTV for the SRS plans measured using the CC01.

**Fig. 5:**
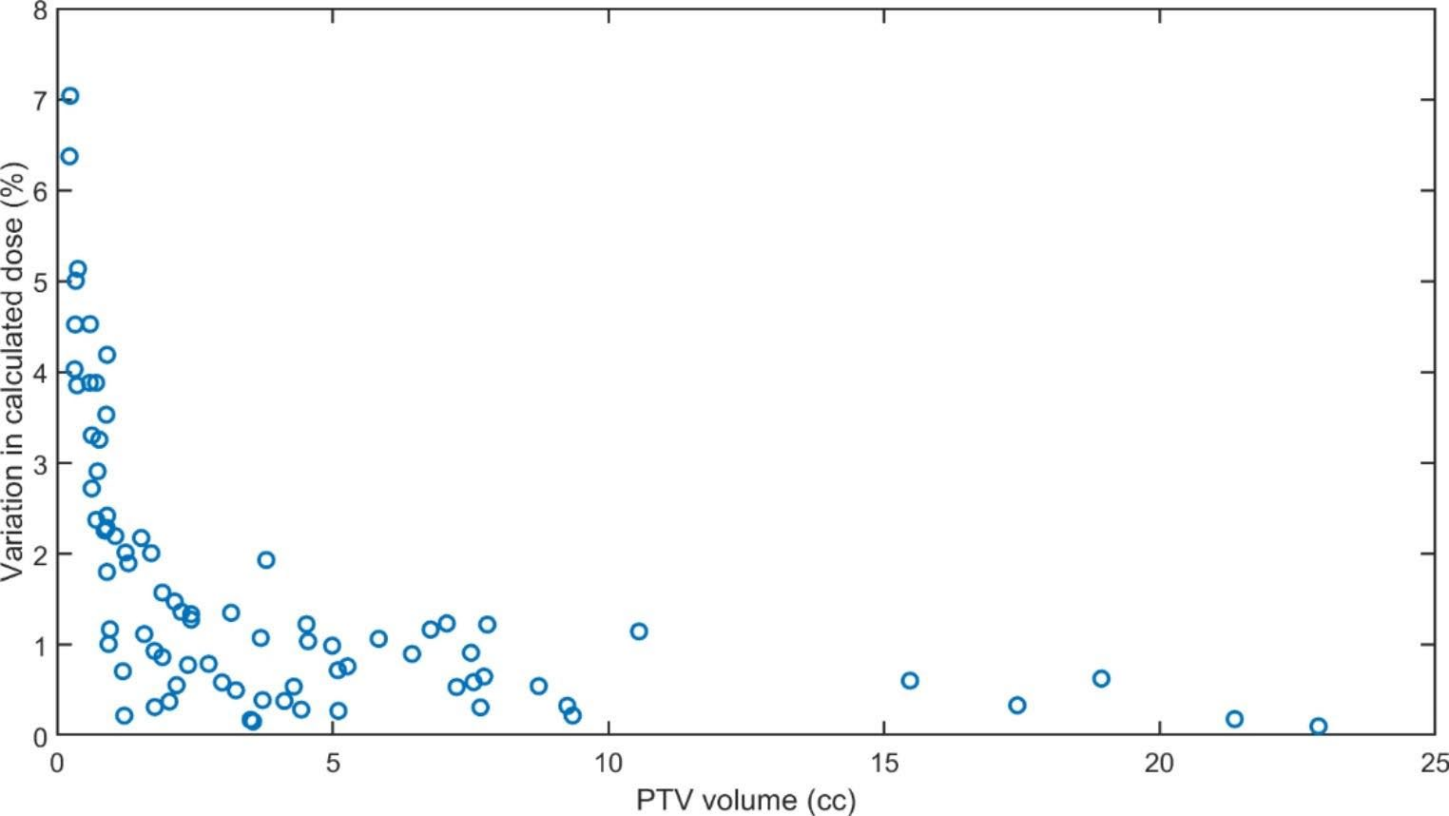
The variation in calculated dose across the range of considered sphere sizes and chamber models with volume of the clinical PTV

Three larger volume treatments were excluded from the plot due to scaling. Each showed a variation in calculated dose of less than 0.7%. It can be seen that very small PTV are more likely to give rise to higher variations in the calculated dose although there are PTV with very small volume and very small variation. From Fig. 5 it is suggested that PTV under 5 cc and 2.5 cc are susceptible or very susceptible to variations in calculated doses of over 1.0% and an assessment of the most appropriate value should be made by the clinical physicist for the plan under consideration.

## Discussion

A series of correction factors for use with small field dosimetry commissioning within a MC TPS have been presented along with a measure of their uncertainty and therefore applicability. In addition, an approach for obtaining an optimal spherical VOI to represent ionisation chamber expected dose during PSQA was presented. The values obtained using this approach, which consequently require no additional correction factors during measurement, were evaluated against previously measured PSQA results.

### Correction factor values and uncertainty

When selecting the most suitable correction factor, the aim should be to use a minimal value which still produces a result with an acceptable uncertainty. For field sizes of 8 mm and above, factors of less than 5% were obtained when accepting a maximum of 1% as two standard deviations of the errors in corrected point doses. This indicates that these factors could be used during commissioning, falling within the 5% maximum value recommended for measurements based corrections. [3].

For smaller field sizes of 5 and 6 mm, corrections factors of up to 8.4% and 6.7% would be required, respectively, to maintain two standard deviations of corrected error at 1%. It is however important to recognise this radius would encompass the majority of the open field and the correction factors for this radius well exceed 5%. These correction factors should therefore not be used in a commissioning process, instead, more histories should be used in the calculation if possible. [8] Alternately, a larger uncertainty in the correction factor could be accepted. Factors of less than 5% could be extracted for 5 and 6 mm fields if acceptable values of uncertainty were set as 1.5% and 1.3% respectively. Ideally, these values could be used in combination with calculations on a small number of phantoms to allow further averaging and uncertainty minimisation. It is also important to remember the finite voxel size with the treatment planning which results in volume averaging over a reasonable proportion of the open field size, even when using the smallest sphere size possible within Monaco.

For the smallest tested field size of 4 mm a minimum of 1.1% was found as two standard deviations of the error over corrected doses. While this might be reasonable for commissioning such small fields, it would require correction factors in excess of 10%, which are not reasonable. In Fig. 2 the value of two standard deviations can be seen to increase again for spheres of radius over 2 mm due to the complete encapsulation of the field and significant contributions of out of field modelling dose. For these smallest of field sizes, correction factor-based approaches can be considered unreasonable. Care should be taken in determining dose for such small fields when using a MC calculation system with a finite voxel size, and the optimal approach would be to repeat the calculation over a number of phantoms.

When considering field sizes of 8 mm and above, a range of correction factors are presented with variable magnitude and uncertainty. The commissioning team could therefore decide on the most suitable to their application. It should be noted however, that reduction in uncertainty is minimal for sphere radii of greater than 1.8 mm suggesting that larger sphere radii offer little benefit in exchange for increased magnitude in the factor.

The similarity of the factors (presented in appendix 1) for the 6MV and 6FFF beam models suggest that the presented average values are not highly specific to the clinical models for the investigating institution. While care should be taken to consider the relevance in new clinical environments, the use of generic factors is similar to the approach of the IAEA factors, [3] also given as only a single set of values for 6MV or 6FFF.

### Sphere radius association with ionisation chambers and PSQA

To obtain the best level of agreement between the correction factors presented by the IAEA and those established in this study, [3] volumes greater than the active volume of the investigated ionisation chamber were required. While the factors presented from this investigation are purely volume averaging factors, those presented by the IAEA account for chamber performance in addition, including consideration of the materials of construction. As such they may not be expected to exactly match.

Composite plan PSQA calculations showed variation of less than 1% in the median dose across all sphere sizes when considering 82 SRS treatment plans, and less than 0.3% across 28 SBRT plans. This strongly indicates that the choice of sphere size is not a crucial consideration in the PSQA process. For the treatment plans where the calculated dose varied by greater than 1% the optimal agreement between the CC01 and calculated dose was observed for a sphere radius of between 1.5 and 1.8 mm. This was the radius range which also showed the greatest agreement with the IAEA factors when calculating small field sizes, suggesting the method for determining the optimal spherical volume holds true under PSQA practice and could potentially be extended to additional chambers or detectors.

The model of the CC01 considered in this study showed a very similar level of agreement with PSQA measurements as the 1.5 and 1.8 mm spheres, and only a slightly reduced agreement with the IAEA factors. This could be related to the limited accuracy in contouring the small ionisation chamber active volume against a dose grid with relatively large voxels, the variation in shape between a spherical volume and the shape of the active volume of the CC01, or could be a reflection of the additional influences the IAEA factors correct for. The CC01 contains a steel electrode in order to increase the signal produced, [13] which may account for the greater volume of averaging required in the TPS, but the same is not true for the CC04 which also required averaging over a larger volume than expected.

From these results it can be suggested that either a sphere radius of 1.5 to 1.8 mm or a CC01 active volume contour could be used for optimal agreement between TPS calculated composite dose PSQA measurements. The use of a small sphere has benefits when using the Monaco TPS due to its easy implementation and repeatability about a precisely known location in contrast to a contour of a CC01 active volume which requires manual contouring and results in voxel ownership associated with the CT voxel information and not the dose grid. [17] For each CT scan the contouring of a chamber volume would therefore require careful consideration.

For the SBRT plans measured using a CC04, negligible variation was observed in the calculated dose across the full range of sphere radii considered. Unlike the CC01, no notable change in the level of agreement between PSQA measurement and the sphere size which showed the best agreement when compared to the IAEA factors was therefore observed. This may contrast with expectation; one may reasonable expect that using a sphere of volume greater than one and a half times the active volume of the chamber could result in reduced agreement due to a greater level of volume averaging in the TPS. As a result, the methodology presented here to determine the optimal sphere radius for the CC01 could not be confirmed for the CC04. However, it was shown that using the larger recommended volume did not decrease the agreement. This allows physicists to confidently use a larger volume and minimise the risk of effects due to statistical noise, although they are unlikely to arise in practice.

### Identification of sensitive treatment plans

The present study has shown that in clinical situations the choice of VOI is not a major contributor to uncertainty in the PSQA process but can have significance for the smallest of PTV, under 5 cc. If the target is smaller than 5 cc, the range of doses to a number of small VOI should be considered to determine the most applicable value to be compared to during measurement, or to adjust the PSQA tolerance accordingly.

### Additional considerations

The presented correction factors were derived using the Monaco TPS and are therefore primarily intended for use within Monaco. Care should be taken if extrapolating use to another MC system. Monaco uses variance reduction techniques and voxelisation which may not directly translate to other systems or TPS. [8,19]

Comparing the results of PSQA to calculations in the TPS to determine or validate the choice of an optimal volume to represent an ionisation chamber assumes no systematic error in the TPS calculations or PSQA measurements. These risks were minimised in this study by collecting the PSQA data on multiple linacs, delivered by different physicists, for measurement of PSQA and the use of correction factors which are a function of field size only and would not be subject to error in the absolute dose calculation. It is also important to note that although the IAEA provides correction factors for the CC01 chamber, [3] other publications recommend it is not used due to the steel electrode. [13] In this investigation a high level of agreement was observed between the TPS and CC01 measurements over a large number of PSQA measurements, suggesting the use of the chamber is possible under the correct conditions and when using adequate corrections. It would be recommended that in line with other guidance, [13] the CC01 not be used as the sole method of PSQA. For accurate evaluation of such dose distributions, where steep dose gradients are of importance, additional measurements of composite dose delivery using two or three dimensional arrays offer useful additional information. [14].

## Conclusions

For fields sizes of 8 mm and greater a series of correction factors have been presented which led to agreement of the TPS calculated and measured isocentre dose within 1% for over 95% of calculations. For smaller field sizes, correction factors of greater than 5% were required and this approach is therefore not recommended. Instead, accurate determination of isocentre doses for such small fields requires a decreased statistical uncertainty via increased histories or repeat calculations of small fields using a series of phantoms if using a system such as Monaco.

Sphere sizes of approximately double (in voxel space) the sensitive volume of CC01 and CC04 chambers were shown to be required if the IAEA measurement correction factors were to be applied to doses averaged over a VOI within the TPS. As such it is recommended that measurements of composite SRS or SBRT treatment plans be compared to the average dose to a sphere of radius 1.5 to 1.8 mm if using a CC01 or 2.5 to 2.9 mm if using a CC04 ionisation chamber. On review of 82 SRS treatment composite plan PSQA measurements using a CC01 a radius of 1.5 mm was shown to provide optimal agreement. A review of 28 plans measured using a CC04 showed that the choice of sphere size made negligible difference to the predicted dose and larger sphere radii of 2.5 to 2.9 mm were acceptable, minimising any possible, albeit unlikely, statistical noise variations. The process used in this study to determine the optimal radius can be extended to TPS in other clinical departments and may lead to spherical VOI, or chamber delineations, that differ in size to the physical dimensions of the sensitive volume of the ionisation chamber itself. The choice of sphere size has also been shown to be a necessary consideration only for clinical target volumes of less than 5 cc.

## Appendix 1

Full tabulation of correction factors